# Amiodarone Administration during Cardiopulmonary Resuscitation Is Not Associated with Changes in Short-Term Mortality or Neurological Outcomes in Cardiac Arrest Patients with Shockable Rhythms

**DOI:** 10.3390/jcm13133931

**Published:** 2024-07-04

**Authors:** Nicolas Kramser, Dragos Andrei Duse, Michael Gröne, Bernd Stücker, Fabian Voß, Ursala Tokhi, Christian Jung, Patrick Horn, Malte Kelm, Ralf Erkens

**Affiliations:** 1 Department of Cardiology, Pulmonology and Angiology, Medical Faculty, Heinrich Heine University of Düsseldorf, 40225 Düsseldorf, Germany; 2 Cardiovascular Research Institute Düsseldorf (CARID), 40225 Düsseldorf, Germany; 3 Department of Cardiology and Electrophysiology, St. Agnes-Hospital Bocholt, 46397 Bocholt, Germany

**Keywords:** Amiodarone, cardiopulmonary resuscitation, survival, neurological outcomes

## Abstract

**Background:** The search for the best therapeutic approach in cardiopulmonary resuscitations (CPR) remains open to question. In this study, we evaluated if Amiodarone administration during CPR was associated with short-term mortality or neurological development. **Methods:** A total of 232 patients with sudden cardiac arrest (CA) with shockable rhythms were included in our analysis. Propensity score matching based on age, gender, type of CA, and CPR duration was used to stratify between patients with and without Amiodarone during CPR. Primary endpoints were short-term mortality (30-day) and neurological outcomes assessed by the cerebral performance category. Secondary endpoints were plasma lactate, phosphate levels at hospital admission, and the peak Neuron-specific enolase. **Results:** Propensity score matching was successful with a caliper size used for matching of 0.089 and a sample size of *n* = 82 per group. The 30-day mortality rates were similar between both groups (*p* = 0.24). There were no significant differences in lactate levels at hospital admission and during the following five days between the groups. Patients receiving Amiodarone showed slightly higher phosphate levels at hospital admission, while the levels decreased to a similar value during the following days. Among CA survivors to hospital discharge, no differences between the proportion of good neurological outcomes were detected between the two groups (*p* = 0.58), despite slightly higher peak neuron-specific enolase levels in CA patients receiving Amiodarone (*p* = 0.03). **Conclusions:** Amiodarone administration is not associated with short-term mortality or neurological outcomes in CA patients with shockable rhythms receiving CPR.

## 1. Introduction

Sudden cardiac arrest (CA) is a leading cause of death worldwide [1], accounting for up to 20% of deaths in Western societies [2]. Despite significant improvements in cardiopulmonary resuscitation (CPR) algorithms and post-resuscitation care, mortality post-CA remains high, with mortality rates as high as 90% for out-of-hospital cardiac arrest (OHCA) [3] and 75% for in-hospital cardiac arrest (IHCA) patients [4]. In surviving patients, only a minority display good neurological outcomes [4]. Several individual traits during CPR, such as time to ROSC, initial rhythm, cause of CA, or bystander CPR, increase the chance of survival and, in survivors, the good neurological outcomes [5,6,7]. Interestingly, most of the characteristics that influence the outcomes seem to be those of the initial presentation of the CA, reinforcing the idea that the preclinical care of the patients is, in fact, the most essential part of the treatment [8]. Therefore, treatment algorithms rely heavily on initially rapidly available and cost-effective markers. Lactate and phosphate are feasible laboratory markers used at bedside to assess disease severity and, in the absence of more precise prognostication markers, to forecast survival chances [9,10,11,12]. In the current era, the use of digitalization, data extraction, artificial intelligence, and machine learning algorithms has already provided accurate risk score models such as the SCARS model [13,14,15,16]. Very interestingly, most of these models incorporate traits from initial presentation and prehospital intervention (i.e., accounting for >55% of the relative importance of the SCARS model [13]), further validating the importance of structured preclinical care.

Unlike factors that cannot be directly influenced, CPR treatment algorithms have been subjected to numerous changes following hundreds of studies searching for the best therapeutic approach. In shockable rhythms, i.e., ventricular fibrillation or tachycardia (VF/VT), the European Resuscitation Council Guidelines recommend using Adrenaline and Amiodarone after the third unsuccessful electrical shock [17]. In the PARAMEDIC2 trial, Adrenaline improved 30 days survival but not the neurological outcome [18]. Amiodarone blocks the potassium currents and modulates sympathetic activity due to interactions with α and β receptors supporting VF/VT termination [19]. In CPR, Amiodarone influences the response to defibrillation when administered for VF or hemodynamically unstable VT [20,21,22].

In older randomized controlled trials (RCT), Amiodarone improved survival chances to hospital admission, proving superior to Lidocaine in terms of survival to hospital admission [23,24]. In newer RCT with OHCA patients suffering from VT or pulseless ventricular tachycardia, neither Amiodarone nor Lidocaine improved survival or neurological outcomes [25,26,27]. Based on these and other trials, Amiodarone administration remains an IIb recommendation and, therefore, an individual physician choice [28]. While these studies displayed the effects of solitary Amiodarone administration, the current guidelines recommend its use along with Adrenaline. Some recent trials have reported a benefit from early Amiodarone administrations in OHCA patients with shockable rhythms [29,30]. However, the full extent of Amiodarone administration supplementary to Adrenaline during CPR on mortality and neurological outcomes, as occurring in a real-world setting, remains unknown. Therefore, the current study aimed to examine whether Amiodarone administered along with Adrenaline in shockable heart rhythms was associated with lower in-hospital mortality and improved neurological outcomes.

## 2. Methods

### 2.1. Study Setting and Population

This study retrospectively analyzed IHCA and OHCA patients who received CPR and were ultimately admitted to the internal medicine Intensive Care Units of the University Hospital of Duesseldorf (UKD) between 2013 and 2017. The database had been previously presented extensively by us [11]. The analyzed data covered the report of the emergency physician and all data documented at the hospital, including laboratory parameters, medical imaging, and documentation until discharge from the hospital. Pre-existing illnesses were segmented into cardiac (heart failure, cardiomyopathies, coronary heart disease, valvular diseases) and pulmonic diseases (chronic obstructive pulmonic disease, bronchial asthma, lung cancer, pulmonic hypertension).

Initially, all patients were treated in accordance with the guidelines applicable at the time for IHCA and OHCA [31,32]. All patients received Adrenaline and one or more defibrillation attempts by electrical shock. Following ROSC, the patients were admitted to the non-surgical intensive care unit of the University Hospital of Duesseldorf, where target temperature management with a target of 34 °C was established for 24 h, followed by 72 h of normal body temperature.

Group stratification was according to Amiodarone administration. Exclusion criteria were non-shockable rhythms, patients with incomplete data sets, or patients younger than eighteen years. The primary endpoints of this study were the 30-day mortality and neurological outcomes among survivors until hospital discharge. We used the cerebral performance category (CPC) to evaluate the neurological outcome. The CPC is a five-point scale used to categorize the neurological outcome after cardiac arrest, ranging from good cerebral performance (1) to brain death (5) [33]. It is dichotomized, with 1–2 as good and 3–4 as bad neurological outcomes [34]. Secondary endpoints were the plasma lactate and phosphate levels at hospital admission, the peak neuron-specific enolase (NSE), and cranial imaging results, which were assessed by computed tomography or cranial magnetic resonance imaging.

This study was conducted according to the guidelines of the Declaration of Helsinki and approved by the Medical Faculty of the Heinrich-Heine-University Ethics Committee in Duesseldorf, Germany, with the reference number 2018-109-RetroDEuA. Due to the retrospective nature of our study, informed consent was waived.

### 2.2. Recording of Data

Data were extracted from the hospital information systems *Medico* (Cerner GmbH, Berlin, Germany) and *PEGASOS* (Nexus/Marabu GmbH, Berlin, Germany). For data acquisition, we analyzed all existing physician letters, clinical investigations, the patients’ documentation curves, and laboratory values. Data on the patients’ course before hospitalization were obtained from the ambulance call report, which included documentation of the initial and all subsequent cardiac rhythms during treatment, the drugs administered, and the duration of resuscitation.

### 2.3. Propensity Score Matching

Propensity score matching (PPM) was performed using a logistic regression model. The treatment variable was the administration of Amiodarone during CPR, which acted as the dependent variable. Covariates were age, gender, type of cardiac arrest, and duration of CPR, which acted as independent variables. Propensity scores were calculated using logistic regression. Patients were matched using the nearest neighbor matching algorithm. The maximum caliper size was set at 0.5 and iteratively adjusted to ensure both treated and control groups had at least 75 patients each. The final caliper size used was approximately 0.089. All analyses were performed in R-Studio with ‘MatchIt’, ‘tidyverse’, ‘openxlsx’, and ‘cobalt’ packages.

### 2.4. Statistics

Continuous data are presented as mean ± standard deviation (SD), and categorical data as absolute numbers and percentages. Data were compared with the Student’s *t*-test for normally distributed continuous variables and the Mann–Whitney test for non-normally distributed continuous variables. The Chi-square test was used for categorical data. Differences in survival of the groups were assessed by Kaplan–Meier analysis with Log-rank (Mantel–Cox) test. *p*-values < 0.05 were considered as statistically significant. Statistical analyses were performed using Prism 9.3.0 (GraphPad Software, San Diego, CA, USA). Propensity score matching was performed in R-Studio (R version 4.1.2 (2021-11-01), R Foundation for Statistical Computing, Vienna, Austria).

## 3. Results

### 3.1. Study Patients

**Figure 1:**
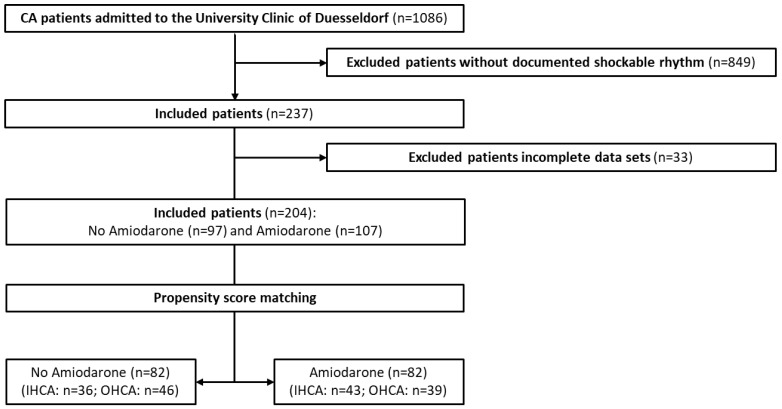
Flow chart of the CA patients’ population in our analysis.

Between 2013 and 2017, 1086 patients with cardiac arrest were admitted to the Intensive Care Units of the University Hospital of Duesseldorf. Of these, 237 patients presented a shockable initial heart rhythm. To achieve robust PPM, all patients without documented characteristics used for matching (i.e., age, gender, type of CA, CPR duration) were excluded from analysis (*n* = 33). The remaining 204 patients were used for PPM (Figure 1).

PPM was successful with the final caliper size used for matching of 0.089, resulting in two groups of *n* = 82 being created. The overlap coefficient was 0. The standardized mean differences (Table 1) highlighted balanced comparison groups with substantial improvements after matching. In addition to the variables used for matching, there were no significant differences in CPR cause, it being primarily cardiac in both groups (*p* = 0.86). Notably, the returns to spontaneous circulation rates were nearly equal (84% in patients without Amiodarone versus 83% in patients receiving Amiodarone, *p* = 0.83). The complete baseline comparison between the groups is shown in Table 2.

### 3.2. Amiodarone Administration during CPR Is Not Associated with Short-Term Mortality

**Figure 2:**
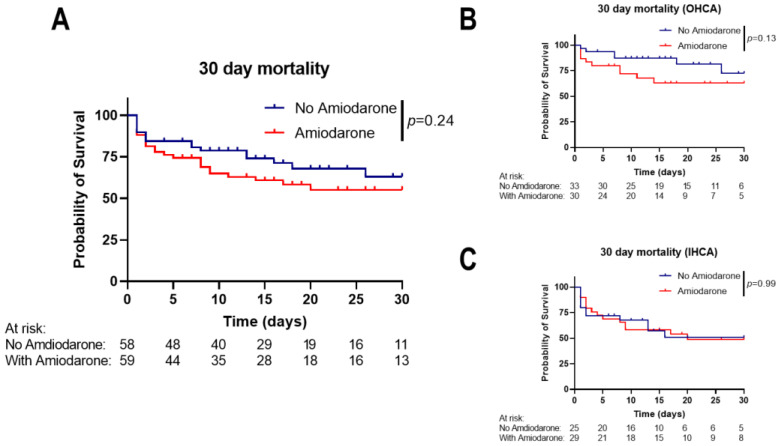
Kaplan–Meier survival analysis within the first 30 days of the entire collective (**A**), OHCA (**B**) and IHCA patients (**C**). No significant difference in the probability of survival between the different groups was detected in the entire collective or stratification according to type of CA. Statistical analysis was performed by Mantel–Cox test. *p*-value as shown in the figure.

**Figure 3:**
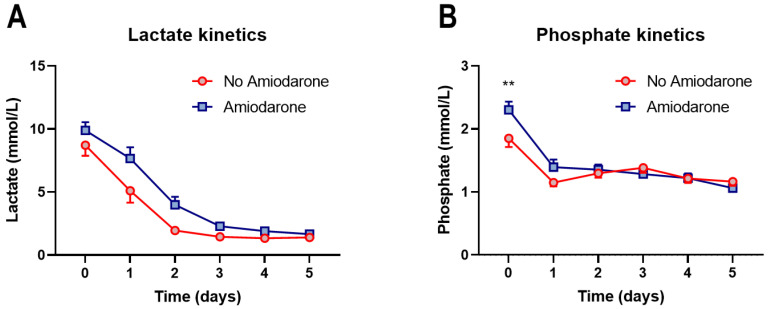
Plasma lactate (**A**) and phosphate (**B**) levels at hospital admission and the following five days after CPR. (**A**) No significant difference between lactate at any time point. (**B**) A mild but significant difference in initial plasma phosphate levels at admission, with similar dynamics over the following five days between the comparison groups. Statistical analysis was performed by two-way ANOVA with Šídák’s multiple comparisons to compare the values at each time point. ** *p* < 0.01.

Comparison of the 30-day mortality between the matched groups showed no significant difference (*p* = 0.24, hazard ratio (Mantel–Haenszel) for administering Amiodarone: 1.46, 95% CI: 0.78–2.74; Figure 2A). A similar 30-day mortality for administering Amiodarone was seen in the subgroups of OHCA and IHCA patients (Figure 2B,C). Lactate levels, which are well-established prognostic markers in CA patients and after CPR [35], were similar from hospital admission and during the following five days of hospitalization (*p*-value for interaction: 0.14, Figure 3A). We recently showed that serum phosphate levels can be used as prognostic markers [11,36]. We saw a slight phosphate level elevation at hospital admission in patients receiving Amiodarone (1.85 ± 0.96 mmol/L vs. 2.3 ± 0.99 mmol/L, *p* = 0.0038), while the levels were similar during the following days between the groups (Figure 3B). In a Cox regression analysis adjusted for initial lactate and phosphate levels, administering Amiodarone was not significantly associated with survival to hospital discharge (Hazard Ratio: 1.23, 95% CI: 0.67–2.32, *p* = 0.52).

### 3.3. Amiodarone Administration during CPR Is Not Associated with Neurological Development

**Figure 4:**
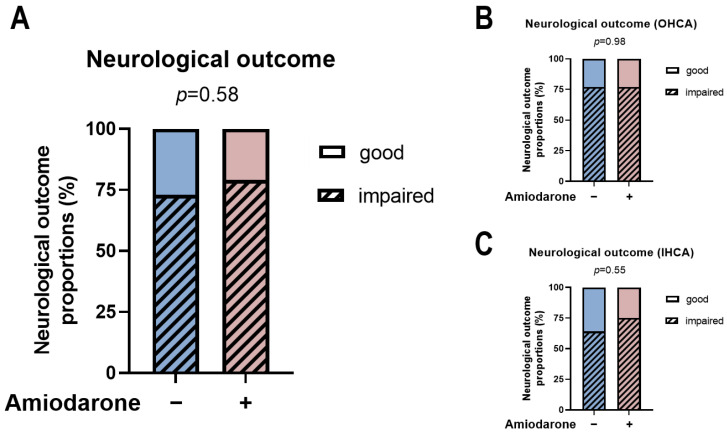
Neurological outcome assessment at hospital discharge using the cerebral performance category in all surviving CA patients (**A**) and stratified according to OHCA (**B**) and IHCA (**C**). No visible difference between any of the groups. Good neurological outcome was defined as a CPC score of 1–2, whereas impaired neurological outcome was assumed for patients with a score of 3–4. Statistical analysis was performed using the Chi-square test. *p*-value as shown in the figure.

**Figure 5:**
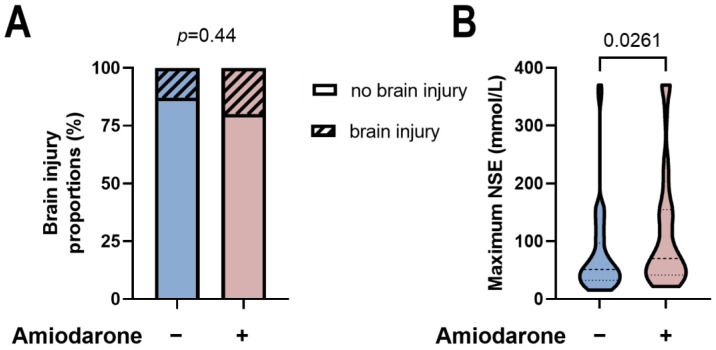
(**A**) There is no difference in brain injury proportions between the two comparison groups. Statistical analysis was performed using the Chi-square test. *p*-value as shown in the figure. (**B**) Higher maximum levels of neuron-specific enolase (NSE) in patients receiving Amiodarone. Statistical analysis was performed using the Mann–Whitney test. *p*-value as shown in the figure.

In survivors to hospital discharge, no difference was detected between initial Amiodarone administration during CPR and later neurological development. There was a proportion of ~20–25% of patients with good neurological development, highlighted by a CPC score of 1–2 in each group, whereas the vast majority suffered impairment of neurological function, characterized by CPC scores of 3–4 (Figure 4A, *p* = 0.58). Subgroup stratification according to the type of CA showed similar results for treatment with or without Amiodarone during the initial CPR (Figure 4B,C). In line, in patients receiving cardiac imaging (i.e., cranial computer tomography or cranial magnetic resonance imaging), the proportion of those with new cranial lesions was similar between the groups (*p* = 0.44, Figure 5A). Despite the comparable outcome rates, there was a higher peak NSE in the group of patients receiving Amiodarone (*p* = 0.0261, Figure 5B).

## 4. Discussion

The current study provides evidence that Amiodarone administration supplementary to Adrenaline during CPR of CA patients with shockable rhythms was not associated with changes in short-term mortality. In addition, analysis of survivors to hospital discharge showed that the initial administration of Amiodarone during CPR had no association with the neurological outcome at hospital discharge. Our analysis was carried out in two subgroups established by propensity score matching and adjusted to resemble each other closely in age, gender, type of CA, and CPR duration, all key factors contributing to prognosis after CPR [5,37]. In fact, newer reports show that the female sex is associated with a lower rate of bystander CPR [38,39], which in turn worsens outcomes of OHCA [3,7]. Similarly, administering Amiodarone during CPR had no link to initial lactate levels, but it might have led to a degree of phosphate and NSE level elevations.

The high mortality and the low rates of good neurological outcomes following CPR highlight the need for research in this field [3,4]. From previous descriptive studies several characteristics are known to be associated with higher mortality. For instance, age and sex play a role in post-CPR development [39,40] but are all individual traits of the patient. The body mass index appears unrelated to OHCA occurrence [41] or following development [42]. CA characteristics such as type of CA, bystander CPR, duration of CPR [43], or initial rhythm are essential factors to consider in the prognostication process; however, they rarely can be improved or changed. In contrast to CA characteristics, much can be improved during the initial treatment; therefore, assessing the optimal strategy is of tremendous importance.

The American Heart Association recommends the consideration of Amiodarone when VF/VT persists after two to three shocks [17]. Amiodarone unfolds its rhythm-stabilizing effect primarily by blocking potassium rectifier currents, which are responsible for heart repolarization. This leads to an increased action potential duration and a prolonged refractory period, preventing recurrent tachyarrhythmias [19]. It is not expected that Amiodarone alone converts VF/VT to an organized perfusing rhythm during CPR, but the central aspect of Amiodarone administration is to facilitate successful defibrillation [28]. Conversely, Amiodarone can block beta-adrenergic receptors, decrease the automaticity of the sinoatrial node, and reduce AV node conduction velocity. This can lead to adverse effects such as bradycardia, hypotension, and the development of Torsade de Pointes [44]. Adrenaline causes vasoconstriction (by α1 receptor stimulation), thereby increasing coronary perfusion pressure. This elevated coronary perfusion pressure enhances the rate of return of spontaneous circulation (ROSC) and survival after cardiac arrest [45,46,47]. However, Adrenaline also stimulates β1 receptors, leading to tachycardia (which increases oxygen demand) and arrhythmias, potentially transitioning ROSC to ventricular tachycardia/ventricular fibrillation (VT/VF) or pulseless electrical activity (PEA), making its influence on long-term outcomes uncertain [48]. Additionally, Adrenaline may reduce microcirculatory flow [49,50], thereby increasing the risk of brain injury [51]. This might explain why even studies showing improved survival to hospital discharge have not demonstrated an improvement in favorable neurological outcomes [52,53]. Due to Amiodarone’s potential hypotensive effect and consequent reduction in coronary perfusion pressure, which could decrease ROSC rates, there has been a prevailing notion that it should be administered after a vasopressor [10,26]. Furthermore, Amiodarone’s anti-tachycardic and rhythm-stabilizing effects [19] complement the effects of Adrenaline after ROSC, leading to a delayed administration strategy for Amiodarone. However, with the advent of new Amiodarone formulations containing captisol instead of polysorbate, which do not induce hypotension, and emerging studies indicating a critical survival benefit with early Amiodarone administration [29,30,54], a new approach may be warranted.

In the Amiodarone in the Out-of-Hospital Resuscitation of Refractory Sustained Ventricular Tachyarrhythmias (ARREST), Amiodarone improved survival to hospital admission compared with a placebo [23]. This was also documented in the Amiodarone versus Lidocaine in the prehospital ventricular fibrillation evaluation (ALIVE) trial [24]. However, there was no improvement in survival to hospital discharge, in line with the 2016 published randomized controlled resuscitation outcomes consortium—Amiodarone, Lidocaine or placebo study (ROC-ALPS) [26]. Newer trials suggested that the timing of Amiodarone administration might affect outcomes. Wissa et al. demonstrated that the time to Amiodarone administration was negatively associated with survival [30]. In a retrospective analysis examining the timing of Amiodarone administration in shock-refractory ventricular tachycardias, better survival rates were documented with early Amiodarone administration [29]. Similar results were observed in a double-blind randomized controlled trial involving 2802 patients, where those who received Amiodarone within the first 8 min had better survival rates to hospital discharge and additionally exhibited better neurological outcomes compared to placebo. No differences were found between Amiodarone administration after the first 8 min and placebo [54]. We did not assess the timing of Amiodarone administration in our study since data collection started prior to these published findings.

In the Amiodarone group, we documented an increased initial phosphate while similar lactate levels started from hospital admission and for the following five days. We and others have shown the predictive values of lactate, its clearance, and phosphate on survival and neurological development in IHCA and OHCA patients and critically ill patients [9,10,11,12,55]. This prediction relies upon an association between lactate and tissue hypoxia, which, although recently debated, can be caused by anaerobic metabolism induced by CA [56,57]. Even sufficient chest compressions during CA cannot sustain a systemic oxygen delivery and lead to hypoperfusion of vital organs [58,59]. Therefore, the increased lactate levels represent more organ damage and can be assumed to have a poorer prognosis [9]. Higher phosphate levels can be documented since CPR leads to hypoxia and, therefore, to cell destruction and dysfunction of the kidneys, resulting in an increased release of intracellular phosphate into the bloodstream and a decreased renal excretion since phosphate is filtered in the glomeruli [60,61]. Very interestingly, recently, we have shown that lactate and phosphate can aid the selection of patients benefiting from extracorporeal mechanical circulatory support [36].

Amiodarone may lead to potential cerebral impairment due to its affinity for the Na^+^/Ca^2+^ exchanger, which plays a protective role against hypoxic-ischemic brain injury with sodium regulation [62]. The blockade of this ion transporter by Amiodarone can cause sodium accumulation in the brain [63,64]. This may be a reason why experimental studies documented an exacerbation of brain injuries and worsened neurological function in mice after the administration of Amiodarone post-hypoxic-ischemic insults [65]. However, this field remains ambiguous since Amiodarone has multiple pharmacological effects that are considered neuroprotective. It blocks calcium channels, which prevent cellular hyperexcitability and ischemic glutamate release after ischemic brain injury [64,66]. Furthermore, it inhibits pro-inflammatory cytokine production and reactive oxygen species [67]. Our data showed similar neurological outcomes in survivors at hospital discharge. Following CPR, current guidelines recommend assessing peak NSE between 48 and 72 h after ROSC [68,69,70]. NSE is a glycolytic enzyme located in neurons and neuroectodermal cells [71], with typically low serum levels. However, damage to neuronal tissue can increase its concentration [72], which makes NSE a viable biomarker to evaluate neurological impairment after CPR. Despite unchanged neurological development, we did see an increase in peak NSE in patients receiving Amiodarone.

Our study has several limitations. Due to its retrospective observational nature, no causal conclusions can be drawn. Additionally, the data for several patients remain fragmentary, especially lactate and phosphate levels. Moreover, while we adjusted for several covariates in preparation of PSM groups, several other characteristics might influence outcomes. Therefore, interpretations of the results should be made carefully. The major advantage of our study is the resemblance of our collective to real-life situations, improving awareness of physician choices in CPR.

Altogether, the data from our study suggest that Amiodarone administration can and should be used since there were no negative associations in terms of outcome. Since randomized controlled trials on CA patients might represent a significant challenge due to the need of fast acting, prospective trials might shed some light onto Amiodarone administration per se and the time to intervention in this setting. From a clinical perspective, CA patients represent a very heterogeneous patient group; therefore, it should remain the individual physician’s decision of whether to administer Amiodarone in an absolute emergency, as nearly every resuscitation shows up in an unplanned and individual setting. Due to this heterogeneity, however, whether Amiodarone administration might be considered before Adrenaline or even during defibrillation remains an open question.

## 5. Conclusions

The current study on a propensity-matched collective of CA patients with shockable heart rhythms who did or did not receive Amiodarone during CPR provided evidence that Amiodarone administration supplementary to Adrenaline, reflecting a real-world setting, was not associated with a difference in mortality. In addition, in survivors until hospital discharge, administering Amiodarone did not affect neurological development. To ensure the highest degree of clarity between the comparison groups, all patients compared received at least one electrical shock, Adrenaline, and the groups receiving or not receiving Amiodarone were adjusted for age, gender, type of CA, and CPR duration, all critical and related to post-CPR outcomes. Future prospective studies should assess the safety of administering Amiodarone earlier in the resuscitation process.

## Figures and Tables

**Table 1:** Summary of standardized mean differences before and after propensity score matching.

	Standardized Mean Differences	
Parameter	Before Matching	After Matching
Type of cardiac arrest	−0.505	−0.176
Age	−0.058	−0.026
Gender	−0.031	−0.031
Duration of cardiopulmonary resuscitation	0.628	0.238

**Table 2:** Patient characteristics.

Patients Characteristics			
Characteristics	No Amiodarone	Amiodarone	*p*-Value
*n*	82	82	
Age (y)	66.7 ± 12.9	66.3 ± 13.5	0.87
Gender [male] (%)	64 (78%)	63 (77%)	0.85
Pre-existing illness (%)			
Cardiac disease	64 (78%)	63 (77%)	0.85
Pulmonic disease	7 (9%)	16 (20%)	0.04
Renal disease	17 (21%)	15 (18%)	0.69
Neurological disease	6 (7%)	7 (9%)	0.77
Malignancy	8 (10%)	4 (5%)	0.23
No known illness	10 (12%)	12 (15%)	0.64
Witnessed arrest (%)	58 (71%)	75 (91%)	0.0007
Bystander CPR (%)	13 (16%)	15 (18%)	0.67
CPR cause (%)			0.86
Cardiac cause	62 (76%)	61 (74%)	
Other cause	20 (24%)	21 (6%)	
CPR duration (mins)	28 ± 29	36.6 ± 35	0.09
Location of cardiac arrest (%)			0.27
IHCA	46 (56%)	39 (48%)	
OHCA	36 (44%)	43 (52%)	
Return of spontaneous consciousness (%)	69 (84%)	68 (83%)	0.83
Coronary intervention after CPR (%)	36 (44%)	42 (51%)	0.34

## Data Availability

The raw data supporting the conclusions of this article will be made available by the authors on request.
